# Functional Innervation of Guinea-Pig Bladder Interstitial Cells of Cajal Subtypes: Neurogenic Stimulation Evokes *In Situ* Calcium Transients

**DOI:** 10.1371/journal.pone.0053423

**Published:** 2013-01-09

**Authors:** Susannah M. Gray, J. Graham McGeown, Gordon McMurray, Karen D. McCloskey

**Affiliations:** 1 Centre for Cancer Research and Cell Biology, School of Medicine, Dentistry and Biomedical Sciences, Belfast, Northern Ireland, United Kingdom; 2 Neusentis, Pfizer limited, Portway Building, Granta Park, Cambridge, United Kingdom; Universidade Federal do Rio de Janeiro, Brazil

## Abstract

Several populations of interstitial cells of Cajal (ICC) exist in the bladder, associated with intramural nerves. Although ICC respond to exogenous agonists, there is currently no evidence of their functional innervation. The objective was to determine whether bladder ICC are functionally innervated. Guinea-pig bladder tissues, loaded with fluo-4AM were imaged with fluorescent microscopy and challenged with neurogenic electrical field stimulation (EFS). All subtypes of ICC and smooth muscle cells (SMC) displayed spontaneous Ca^2+^-oscillations. EFS (0.5 Hz, 2 Hz, 10 Hz) evoked tetrodotoxin (1 µM)-sensitive Ca^2+^-transients in lamina propria ICC (ICC-LP), detrusor ICC and perivascular ICC (PICC) associated with mucosal microvessels. EFS responses in ICC-LP were significantly reduced by atropine or suramin. SMC and vascular SMC (VSM) also responded to EFS. Spontaneous Ca^2+^-oscillations in individual ICC-LP within networks occurred asynchronously whereas EFS evoked coordinated Ca^2+^-transients in all ICC-LP within a field of view. Non-correlated Ca^2+^-oscillations in detrusor ICC and adjacent SMC pre-EFS, contrasted with simultaneous neurogenic Ca^2+^ transients evoked by EFS. Spontaneous Ca^2+^-oscillations in PICC were little affected by EFS, whereas large Ca^2+^-transients were evoked in pre-EFS quiescent PICC. EFS also increased the frequency of VSM Ca^2+^-oscillations. In conclusion, ICC-LP, detrusor ICC and PICC are functionally innervated. Interestingly, Ca^2+^-activity within ICC-LP networks and between detrusor ICC and their adjacent SMC were synchronous under neural control. VSM and PICC Ca^2+^-activity was regulated by bladder nerves. These novel findings demonstrate functional neural control of bladder ICC. Similar studies should now be carried out on neurogenic bladder to elucidate the contribution of impaired nerve-ICC communication to bladder pathophysiology.

## Introduction

The normal bladder functions of filling and emptying are achieved by integration of complex physiological mechanisms within the cells of the bladder wall and their higher control by the nervous system. Bladder interstitial cells of Cajal (ICC) have been studied over the last decade and it is not yet known how they contribute to normal bladder function or whether they are controlled by nerves. Bladder ICC comprise distinct sub-populations: networks of stellate shaped ICC in the lamina propria (ICC-LP); elongated, branched intramuscular ICC (ICC-IM) and stellate ICC in the inter-bundle space (ICC-IB). Several lines of evidence support communication between bladder nerves and ICC: firstly, immunofluorescent and electron microscopy show that ICC-LP and ICC-IM lie in close proximity to nerves [1]–[4]; secondly, ICC respond to exogenous carbachol or ATP by firing Ca^2+^ transients indicating functional receptor expression [5]–[7]; thirdly, ICC-nerve contacts in normal bladder are reportedly in the region of 20 nm [1], within the range of a neuroeffector junction [8], [9]. These findings imply that neuronal regulation of ICC activity is feasible but there is currently no direct evidence to support their functional innervation. Such evidence is difficult to obtain and to be convincing would need to clearly demonstrate, in tissue preparations, an ICC response to nerve stimulation rather than to exogenous agonists e.g. ATP or acetylcholine which are also released from non-neuronal sources such as urothelial cells [10].

In vitro studies of bladder strips exhibit neurogenic contractile responses to electrical stimulation of intramural nerves [11] as confirmed by sensitivity to the neurotoxin, tetrodotoxin, showing that they were evoked by neuronal action potentials rather than direct electrical stimulation of SMC. The hypothesis that bladder ICC are functionally innervated is attractive, especially as it is established that gut ICC have a functional innervation and play key roles as intermediaries and amplifiers in the relay of signals from nerve to SMC [12]. It is not known whether a nerve-ICC-SMC signalling pathway exists in bladder. Moreover, we do not yet know whether ICC receive direct functional input from intramural nerves.

Bladder dysfunction is underpinned by aberrant signalling in the cellular components of the bladder wall typified both by changes in cell-cell interactions as well as the development of abnormal physiological signalling. The location and cellular contacts of bladder ICC are altered after spinal cord injury (SCI) [1] and bladder outlet obstruction [13] however the impact of this pathology on bladder dysfunction has not been investigated. Progress has been impeded by a lack of evidence supporting functional innervation of ICC in healthy bladders.

The aim of the present study was to address this knowledge gap by determining whether ICC are directly innervated in bladder tissue preparations using *ex vivo* Ca^2+^-imaging experiments. The findings demonstrate that ICC in the lamina propria, detrusor and perivascular ICC are functionally innervated. The implications of this for bladder physiology are discussed.

## Materials and Methods

### Ethical Approval

Bladders were removed from male Dunkin-Hartley guinea-pigs (200–500 g) which had been killed humanely by cervical dislocation. The protocols were in accordance with Schedule 1, Animal Scientific Procedures Act, 1986, UK and were approved by the local ethics committee, Queen’s University Belfast.

### Tissue Preparation

Bladders were opened longitudinally and the mucosa removed by sharp dissection. Mucosal preparations were pinned, urothelial surface downwards, over two parallel silver electrodes embedded in a Sylgard recording chamber and loaded with fluo-4 AM (Molecular Probes, Life Technologies UK; 1–5 µM in 0.03% pluronic) for 30 minutes. Tissues were perfused with HEPES-Krebs’ solution (mM: 125 NaCl, 5.36 KCl, 11 Glucose, 10 HEPES, 0.44 KH_2_PO_4_, 0.33 NaH_2_PO_4_, 1 MgCl_2_, 1.8 CaCl_2_) via a gravity feed system at a rate of 2 ml.min^−1^ at 35°C for at least 20 minutes before recordings. Drugs were applied via the perfusion system. Electrical field stimulation (EFS) was applied via silver electrodes at a supramaximal voltage (40 V) using a Grass stimulator over a range of frequencies (0.5, 2, 10 Hz) for 10 s, pulse width 0.2 ms.

### Fluorescent Calcium Imaging

Tissues were imaged with a Nikon 80 i upright epifluorescent microscope (Nikon UK Ltd., Surrey, UK) using a water dipping objective lens (×60W Fluor NA 1.0). Fluo-4AM was excited with a mercury lamp (Nikon) which was attenuated with neutral density filters to minimize sample photobleaching. Filter sets appropriate for fluo-4 imaging were selected: 465–495 nm excitation, dichroic mirror 505 nm and 515–555 nm emission with the resulting fluorescence imaged with an electron multiplying charged coupled device camera imaging system (DQC-FS, Nikon) and recorded to a personal computer running WinFluor software (v3.2.25, Dr J Dempster, University of Strathclyde). Images were captured at a frame rate of 20 frames per second (20 fps) using 2×2 binning from the software which represented an acceptable compromise between acquisition speed and image resolution.

Tissues were typically imaged for 10 minutes and photobleaching was not a significant problem. Treatment with pharmacological agents including tetrodotoxin (TTX), atropine and suramin (Sigma-Aldrich, UK) was carried out by first recording control EFS responses, applying the drug for 15–20 minutes while not exposing the tissue to the excitation lamp, and then imaging the effect of EFS in the presence of drug.

Preparations demonstrated spontaneous and EFS-evoked changes in intracellular calcium concentration ([Ca^2+^]_i_), termed Ca^2+^-oscillations or Ca^2+^-transients. Off-line analysis involved drawing regions of interest (ROI) on cells and a ROI on part of the image which did not contain active cells so that background fluorescence could be subtracted from all measurements. The background-corrected fluorescence (F) at any time point was normalised to baseline fluorescence (F_0_). F_0_ was calculated as average fluorescence in the cell of interest, during 100 frames when there was no activity. The amplitude (ΔF/F_0_), frequency and duration of events were measured in WinFluor and Clampfit (pClamp, v10.3) and analysed in Microsoft Excel and Prism software (v4.02, Graphpad). Data are expressed as mean ± standard error of the mean (SEM) and tested with the Kolmogorov-Smirnov test for normality. Statistical comparisons were made with the Student’s paired or unpaired t-test as appropriate where data were normally distributed or the Mann-Whitney test for non-normally distributed data, with P<0.05 considered as significant. The number of observations in an experimental group is denoted as ‘n’.

### Immunofluorescence

Several mucosal tissue preparations were processed for immunofluorescence experiments by fixing in 4% paraformaldehyde for 20 minutes, washing in PBS, blocking in 1%BSA and 0.05% Triton-X 100 for tissue permeabilisation before incubation with the ICC marker, anti-vimentin (1∶200, Sigma-Aldrich, v4630) for 24 hours. Unconjugated primary antibody was then removed by washing with PBS before incubation in Alexa 488, chicken anti-goat (1∶200) and DAPI to counterstain the nuclei. After washing in PBS, tissues were mounted on slides and imaged with confocal microscopy (Nikon C1 mounted on an e90i upright microscope. Control tissues were prepared with omission of the primary antibody to control for the specificity of the secondary and with omission of all antibodies to examine tissue autofluorescence. KIT labelling of tissues pre-labelled with calcium indicators was performed by cooling tissues to 4°C for 30 min to inhibit tissue macrophages, blocking with 1%BSA and incubating with anti-KIT (1∶200; Oncogene, PC34) for one hour. Tissues were washed in Hanks solution and incubated with Alexa 594 (1∶200), washed again in Hanks and then viewed with epi-fluorescence microscopy. Controls were carried out in the same way except the KIT antibody was omitted. ICC labelling was not observed in control tissues.

## Results

### Calcium Signalling in ICC-LP

**Figure 1:**
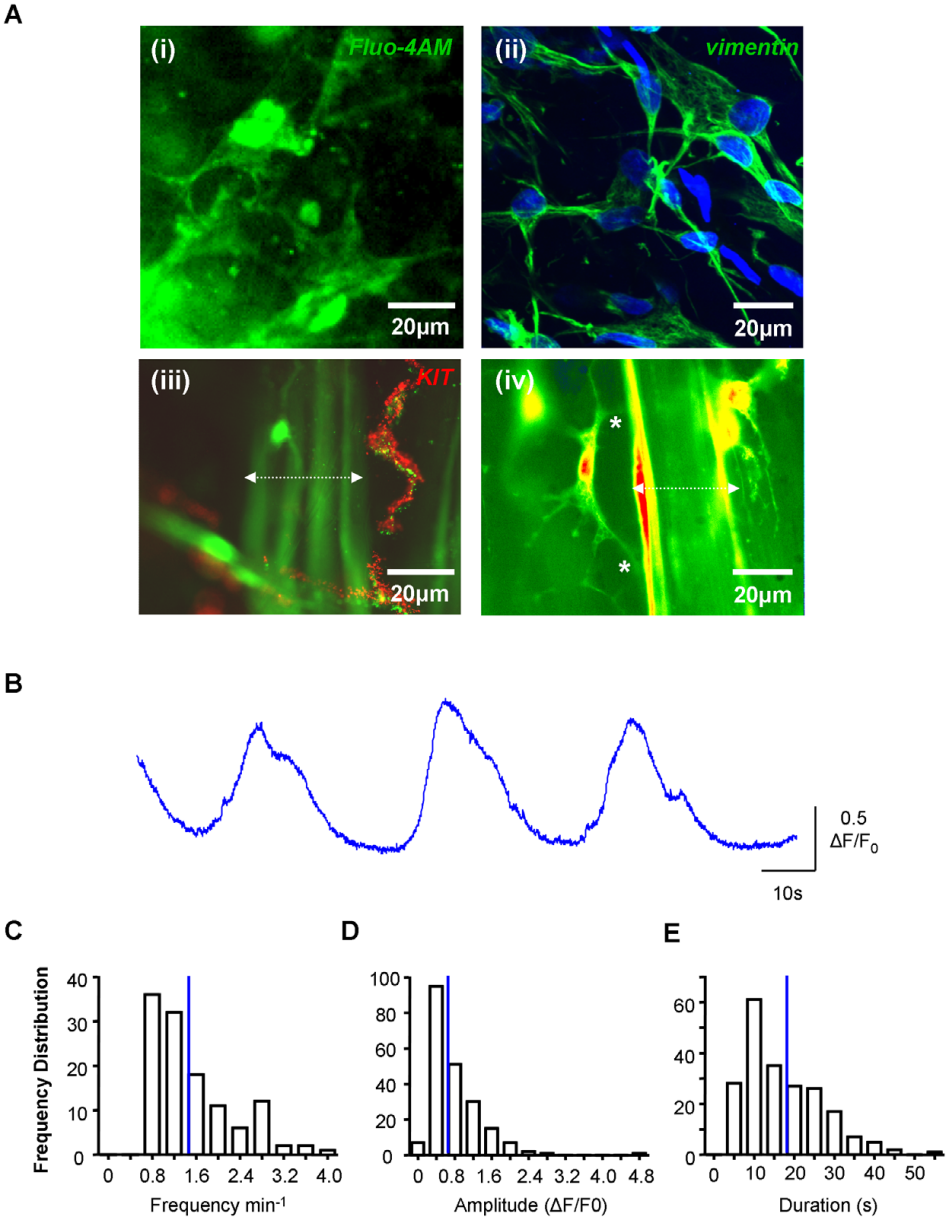
Spontaneous Ca^2+^-signalling in ICC-LP. A. (i) Mucosal preparation of guinea-pig bladder loaded with the calcium indicator fluo-4AM showing networks of lamina propria interstitial cells of Cajal (ICC-LP). (ii) Similar preparation, fixed and labelled with the interstitial cell marker, anti-vimentin. (iii) Preparation loaded with calcium indicator and the ICC marker, anti-KIT showing ICC-IM. (iv) Preparation loaded with calcium indicator showing the structural relationship between ICC-IM and neighbouring smooth muscle. Smooth muscle bundles are indicated in (iii) and (iv) by the dotted white lines. **B.** Typical example of intensity-time series of Ca^2+^-signalling in ICC-LP. **C–E.** Summary data for frequency (n = 120 cells), amplitude and duration (n = 209 events) of Ca^2+^-oscillations in ICC-LP. Mean values are indicated by the blue lines.

Tissues loaded with fluo-4AM contained urothelial cells, microvessels, ICC-LP, ICC-IM and SMC readily identified by their distinctive morphologies when the plane of focus was adjusted. We have previously characterized these cells using antibody markers and confocal microscopy [1], [2], [4], [5], [7]. An ICC-LP network loaded with fluo-4AM is shown in figure 1A along with a similar preparation, fixed and labelled with an ICC marker, anti-vimentin [2]. Smooth muscle bundles and ICC-IM within loaded preparations are shown in figure 1A (iii) where ICC were labelled with anti-KIT. The structural relationship between the branched processes of ICC-IM and neighbouring smooth muscle (figure 1A iv) is demonstrated by removal of neutral density filters from the microscope however; during physiological recordings, filters were used to reduce excitation and minimize phototoxicity. ICC-LP typically exhibited spontaneous increases in [Ca^2+^]_i_ (figure 1B). Frequency distribution plots of the ICC-LP’ spontaneous Ca^2+^ oscillations are shown in figure 1C–E demonstrating mean values and variability in frequency (1.58±0.07 min^−1^, n = 120 cells), duration (17.06±0.67 s, n = 209 events) and amplitude (0.77±0.04 ΔF/F_0_, n = 209 events).

**Figure 2:**
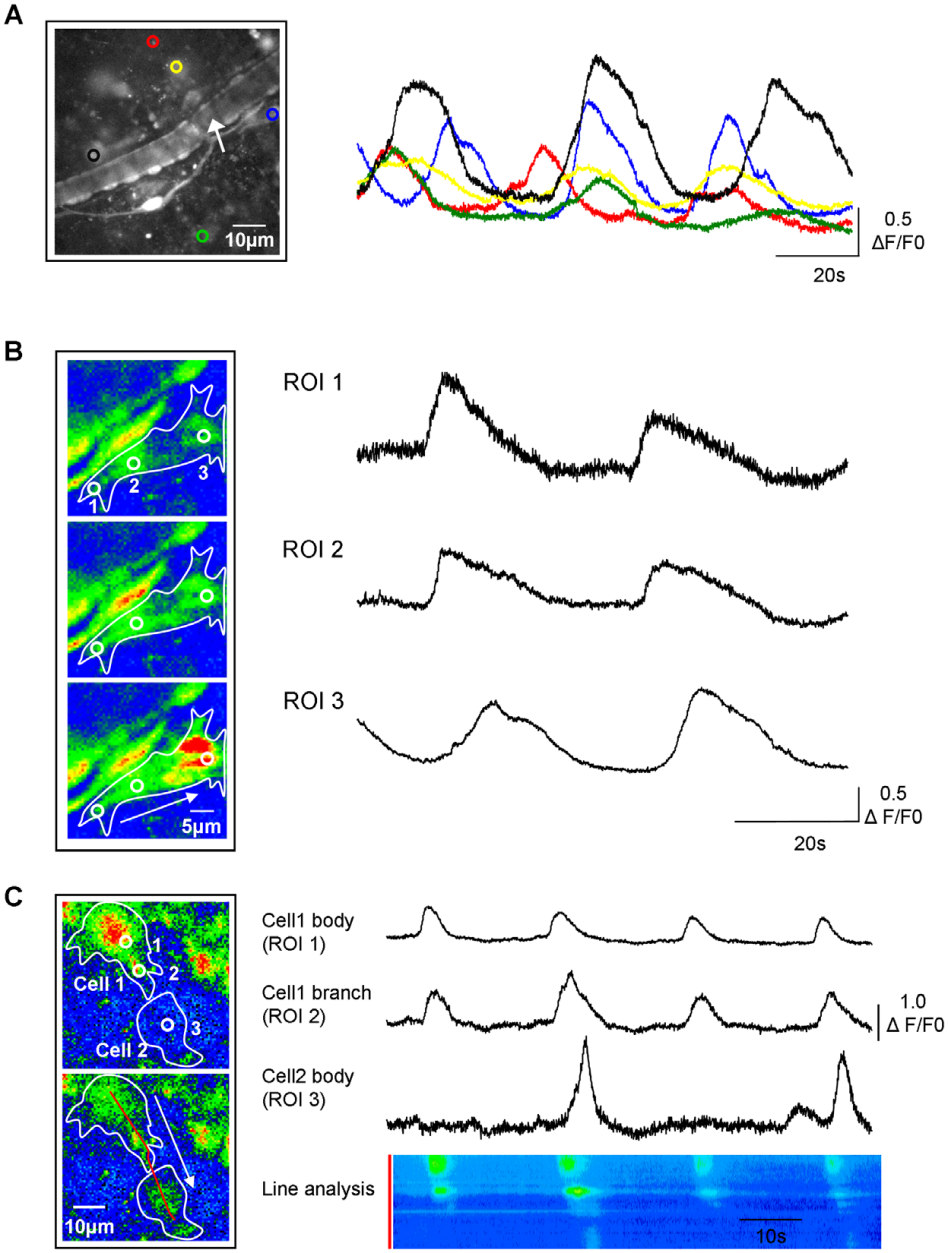
Networked signalling in ICC-LP. A. Low-magnification field of view showing multiple ICC-LP within the tissue and an intensity-time plot of activity in the ICC-LP network indicated by the coloured regions of interest. A typical mucosal microvessel is present in the middle of the micrograph (arrow). **B.** The cell indicated by the blue region of interest in panel A, is shown at higher magnification. Three regions of interest (ROI) indicate the tip and middle of a branch and the cell body and the micrographs show the direction of travel of the Ca^2+^-event from the tip of the branch (ROI 1) through the length of the branch (ROI 2) to the cell body (ROI 3) as indicated by the white arrow. Intensity-time plots for ROI 1–3 show signals travelling within the ICC-LP for 80 s from the tip of the branch to the cell body. **C.** Images of activity in 2 adjacent ICC-LPs showing spread of the signal from the body of cell 1 (ROI 1), along a branch (ROI 2), to the body of cell 2 (ROI 3). Intensity-time plots show that transmission from cell 1 to cell 2 was not always successful (occurring 2 out of 4 times in the example shown). This recording is also illustrated as a post-hoc x-t line analysis where the fluorescence intensity along the red line along cell 1 and cell 2 is plotted against time.

ICC-LP networks are interconnected with Cx43 gap junctions [3], [14]. Here, multiple ICC-LP fired spontaneous Ca^2+^ oscillations at similar frequencies (figure 2A) although they were non-synchronous. Within individual ICC-LP, Ca^2+^ signals were observed to travel from lateral branches to cell body (figure 2B) and also from the branch of one cell to that of a neighbouring cell (figure 2C). However, intercellular transmission was not always successful. This is illustrated both with intensity-time plots from 3 regions of interest and post-hoc x-t analysis of fluorescence intensity along a line drawn through cell 1 and cell 2.

### ICC-LP are Functionally Innervated

**Figure 3:**
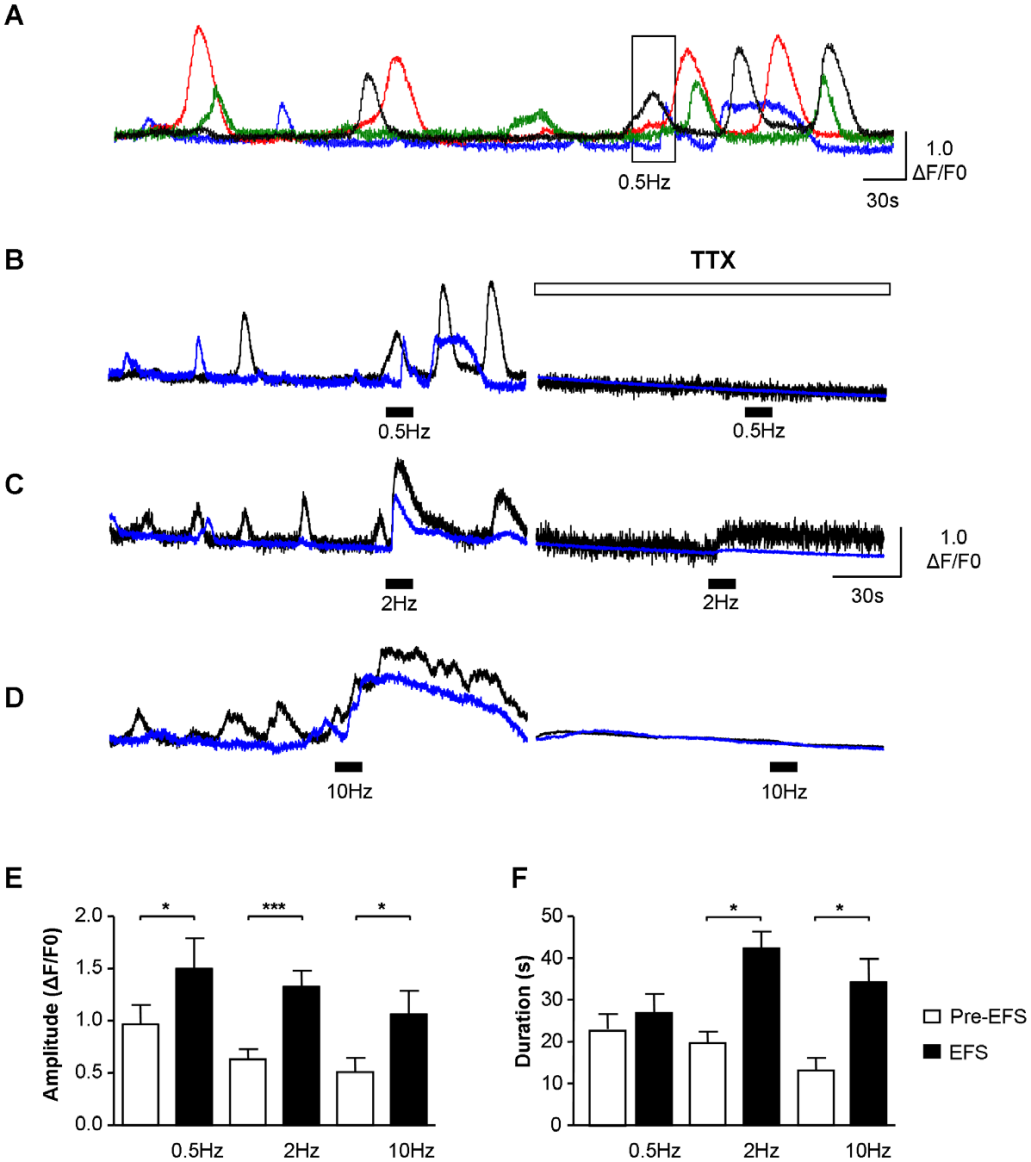
Functional innervation of ICC-LP. A–D. Example of ICC-LP within a field of view responding to 0.5 Hz electrical field stimulation (EFS) where 4 cells were exhibiting non-synchronous Ca^2+^-oscillations pre-stimulation. EFS coordinated the activity of the 4 cells, transiently increasing their frequency of firing but not increasing the duration of the signals. Higher frequencies (2 Hz and 10 Hz) evoked simultaneous, large amplitude, long duration Ca^2+^-transients. EFS responses at the 3 frequencies tested were blocked by the neurotoxin, tetrodotoxin (TTX, 1 µM). (Note that traces from the 2 cells shown in panel B are from the record in panel A with corresponding colours). **E, F.** Summary data for amplitude and duration of pre-EFS Ca^2+^-oscillations and EFS-evoked Ca^2+^-transients, showing a significant increase by EFS (* represents P<0.05, ** represents P<0.01; *** represents P<0.001).

Multiple ICC-LP within a field of view exhibited non-synchronized spontaneous Ca^2+^-oscillations and more than 70% of ICC-LP responded to EFS; 75.5±10.0% at 0.5 Hz, 97.1±1.9% at 2 Hz and 72.4±10.8% at 10 Hz (figure 3A, B, C). The percentage of cells responding to EFS at 0.5 Hz may in fact be higher than 75.5% as it was difficult in some preparations, to separate the EFS response from the background spontaneous oscillations. Similarly, at 10 Hz, the percentage of cells responding could be greater than 72.4% as this stimulation frequency sometimes evoked contraction of the preparation generating a movement artifact which interfered with the EFS Ca^2+^ transient. Low frequency stimulation (0.5 Hz) co-ordinated the signalling within the individual ICC-LP without significantly affecting the duration of the oscillations whereas EFS at higher frequencies (2 Hz, 10 Hz) evoked near-simultaneous Ca^2+^-transients of significantly larger amplitude and duration than the spontaneous oscillations (figure 3D, E). A lag between the start of EFS and onset of Ca^2+^-events was noted at the 3 frequencies tested; 5.41±1.12 s (n = 18 cells) at 0.5 Hz, 5.75±0.83 s (n = 82 cells) at 2 Hz and 3.08±0.72 s (n = 20 cells) at 10 Hz. In the examples shown, ICC-LP which were not synchronized pre-EFS, were notably coordinated by 0.5 Hz and 2 Hz EFS with several cells firing within 2.35±1.13 s (0.5 Hz, n = 3 preparations) and 1.78±0.66 s (2 Hz, n = 5 preparations) of each other. EFS responses were abolished by TTX (1 µM) confirming their neurogenic origin. Interestingly, TTX often suppressed spontaneous Ca^2+^-signalling, suggesting a role for nerves in setting basal activity; this observation was not further pursued but is considered in the discussion section. Comparison of spontaneous and EFS-evoked events demonstrated that the EFS phenomena had significantly different amplitudes and durations at each stimulation frequency than pre-EFS spontaneous events (figure 3D, E).

### Pharmacology of ICC-LP Responses to EFS

**Figure 4:**
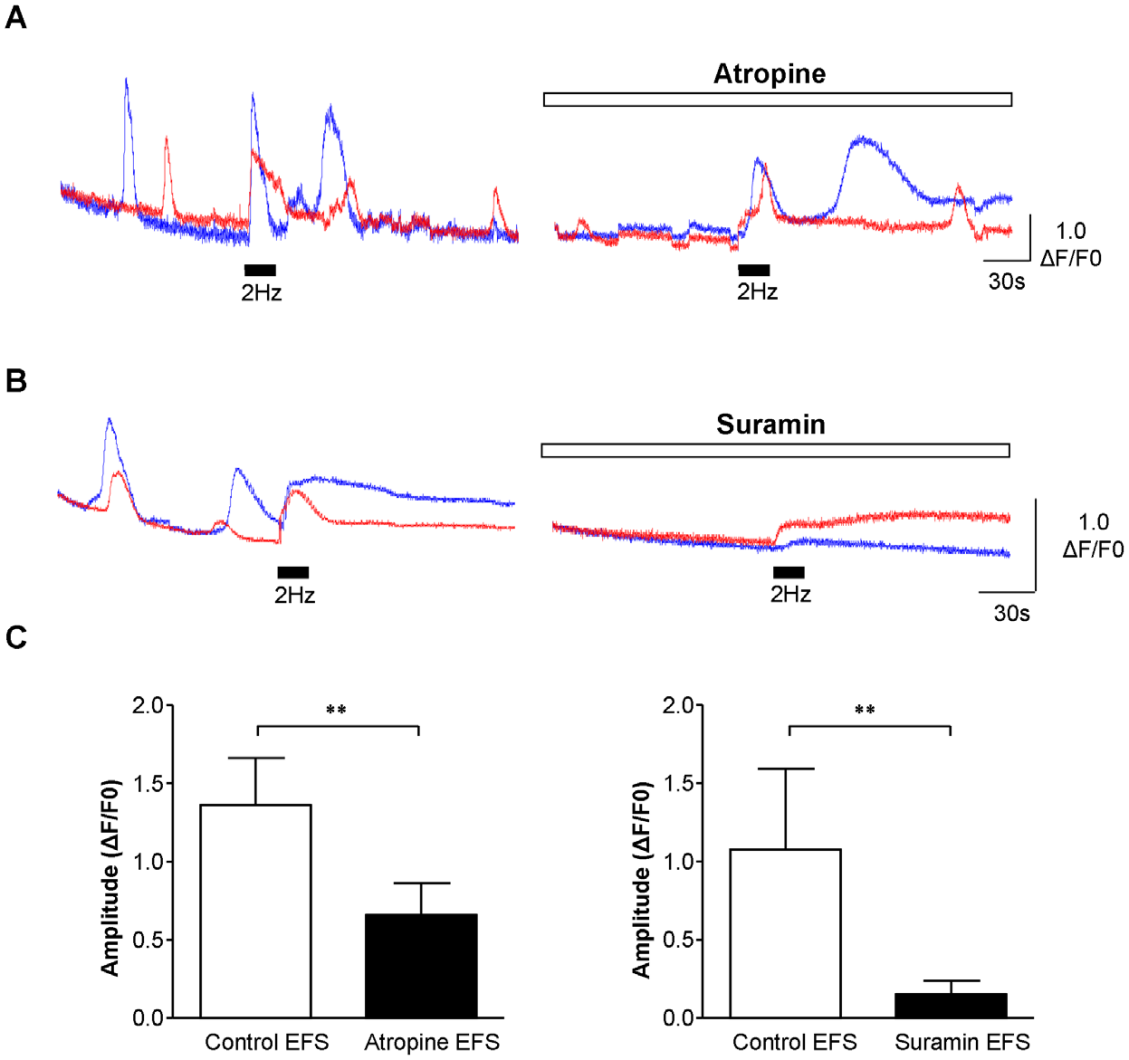
Pharmacology of ICC-LP EFS responses. A. Example from a series of experiments where atropine (1 µM) reduced the amplitude of the response of ICC-LP to EFS (2 Hz). **B.** Similar experiment in a different preparation showing that the ICC-LP response to EFS was reduced by suramin (100 µM). **C.** Summary data showing the significant reduction of the ICC-LP EFS response by atropine in 17 cells from tissues from N = 7 animals (p = 0.0011) or by suramin (p = 0.0301; n = 10 cells from tissues from N = 2 animals).

The EFS responses in ICC-LP were tested with pharmacological drugs to test whether they were mediated by stimulation of cholinergic or purinergic nerves. After EFS responses were recorded in the absence of drugs, the cholinergic receptor antagonist, atropine, or the purinergic receptor antagonist, suramin was added for 15–20 minutes. During application of the antagonist, the microscope shutter was closed to protect the tissue from excessive excitation and to minimize phototoxicity. Atropine or suramin reduced the amplitude of the ICC-LP response to EFS (2 Hz) as shown in the representative experiments in figure 4A and B. Mean data is presented in the bar charts (figure 4C) where responses in n = 17 ICC-LP from tissues from N = 7 animals was significantly reduced by atropine (1 µM; p = 0.0011 Mann-Whitney). Spontaneous activity persisted in atropine although the amplitude of oscillations was often reduced. Suramin (100 µM) significantly reduced EFS amplitude in n = 10 cells from tissues from N = 2 animals (p = 0.0301 Mann-Whitney).

### Effect of EFS on Intramuscular ICC in Mucosal Preparations

**Figure 5:**
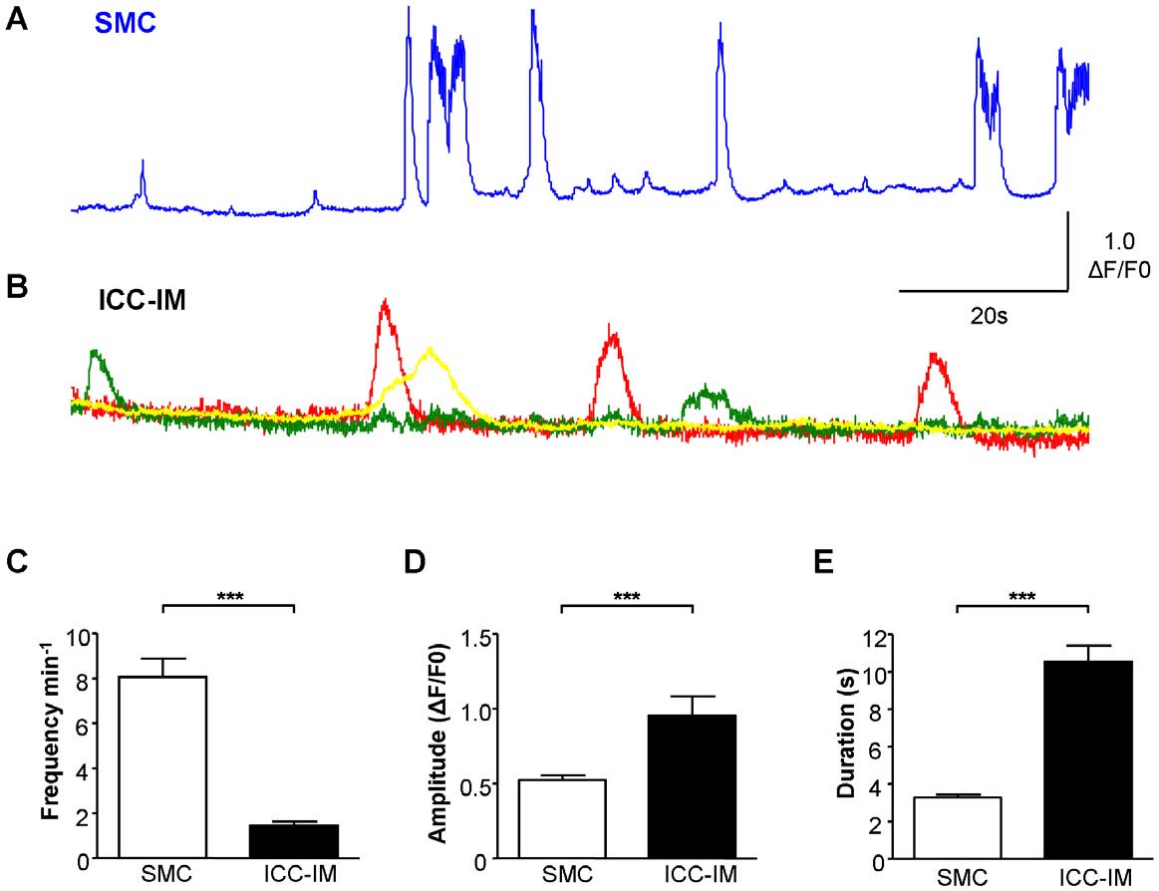
Spontaneous Ca^2+^-oscillations in ICC-IM and detrusor SMC. A. Example of spontaneous Ca^2+^-signalling in bladder smooth muscle (SMC) showing typical large- and small-amplitude events. **B.** ICC-IM (intramuscular ICC) adjacent to the SMC from the same preparation as in A, had a distinctively different pattern of spontaneous activity which was apparently uncorrelated with SMC signalling. **C–E.** Summary data comparing spontaneous Ca^2+^-signalling in SMC and ICC-IM demonstrating the significant differences between the two cell types in frequency (n = 25 SMC, n = 19 ICC-IM), amplitude and duration (n = 325 SMC events and n = 47 ICC-IM events).

**Figure 6:**
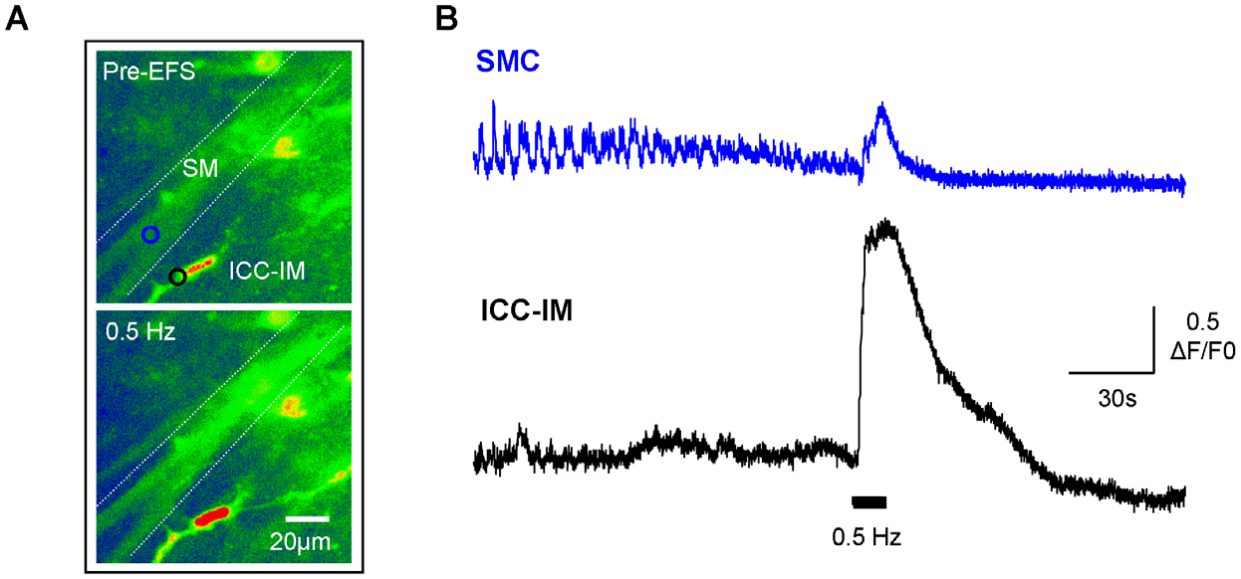
ICC-IM are functionally innervated. A. Micrographs of a fluo-4AM loaded preparation showing a smooth muscle bundle and an associated ICC-IM pre-EFS and during 0.5 Hz EFS. **B.** Intensity-time plot from the 2 regions of interest indicated in F, showing activity in a SMC and an ICC-IM pre-EFS and during EFS (0.5 Hz). The SMC was firing at a higher frequency than the ICC pre-EFS with apparently no correlation between the 2 cell types. Neurogenic stimulation evoked large-amplitude, simultaneously Ca^2+^-responses in both cells.

SMC bundles within the mucosal tissues may have been derived from the muscularis mucosa [15], [16] and/or underlying superficial detrusor bundles. It is difficult to be certain whether the SMC bundles studied were from the muscularis mucosa or superficial detrusor as the muscularis mucosa layer in guinea-pig bladder is discontinuous. Having obtained compelling evidence for functional innervation of ICC-LP, the ICC subpopulation associated with SMC, ICC-IM, were studied with similar protocols. SMC and neighbouring ICC-IM exhibited distinctively different patterns of spontaneous Ca^2+^-oscillations (figure 5A, B) with SMC firing 8.07±0.82 min^−1^ (n = 25) and ICC-IM less frequently at 1.45±0.18 min^−1^ (n = 19; P<0.001 Mann-Whitney). SMC fired small and large amplitude events with mean amplitude and duration of 0.52±0.03 ΔF/F_0_ and 3.27±0.17 s (n = 325 events) respectively, compared with the ICC-IM events of 0.95±0.13 ΔF/F_0_ and 10.51±0.88 s (n = 47 events, P<0.001 Mann-Whitney). Pre-EFS, there was apparently no correlation between oscillations in SMC and neighbouring ICC-IM (figure 5A, B). However activity in the 2 cell types was strikingly synchronized during 0.5 Hz EFS (figure 6 A,B). ICC-IM adjacent to SMC responded to EFS (0.5 Hz or 2 Hz) with an increase in baseline Ca^2+^ or an increase in amplitude and duration of Ca^2+^ oscillations (typical of n = 4 tissues). EFS at frequencies greater than 2 Hz evoked large SMC contractions which made it difficult to visualize any response.

### EFS of Vascular Smooth Muscle and Perivascular ICC

**Figure 7:**
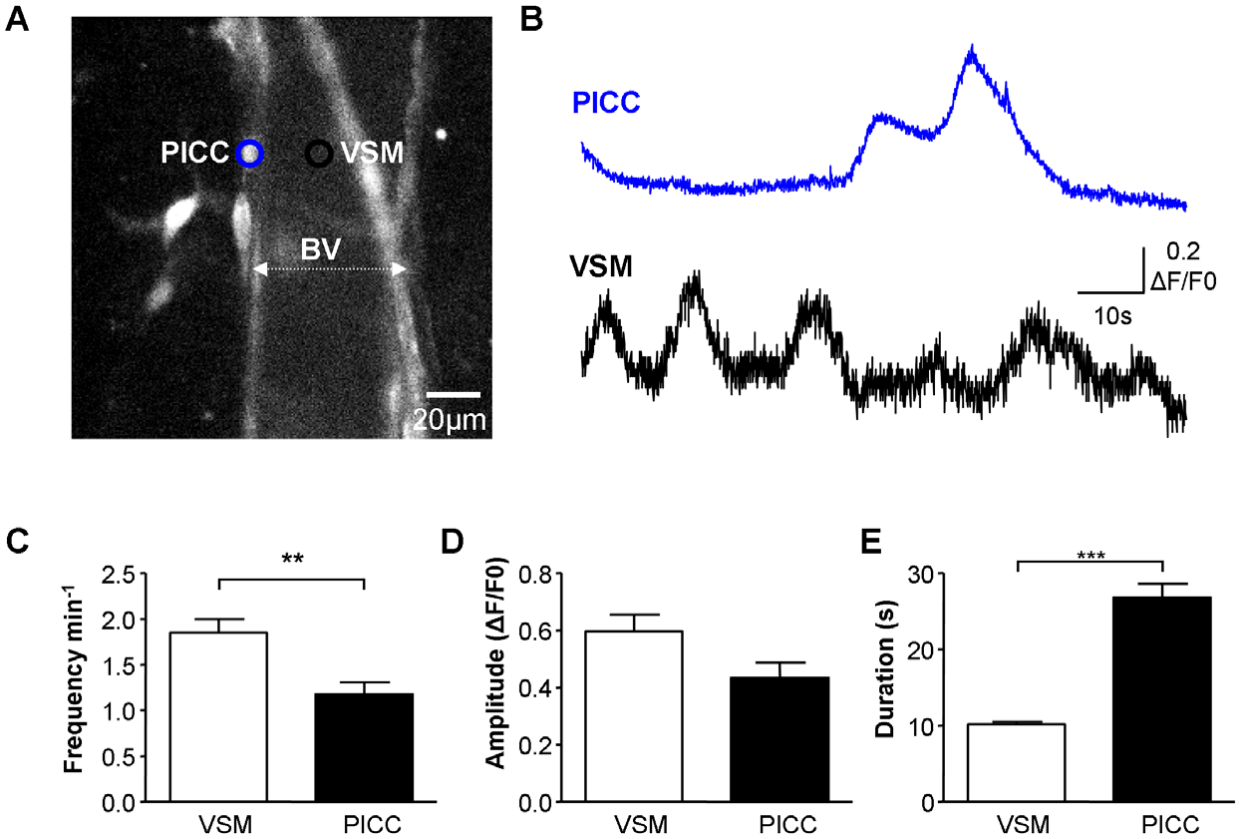
Ca^2+^-oscillations in PICC and VSM. A. Micrograph of a mucosal microvessel shown by dotted white line, and associated perivascular ICC (PICC). **B.** Intensity-time plot of Ca^2+^-activity in the vascular smooth muscle cell (VSM; black region of interest) and associated PICC (blue region of interest) from A. Both cell types showed spontaneous Ca^2+^-activity which was apparently not correlated. **C–E.** Summary data showing significant differences in the frequency and duration of spontaneous Ca^2+^-activity between the two cell types (45 VSM and 21 PICC). PICC had less frequent, longer duration, similar amplitude events in comparison to VSM.

Vascular SMC (VSM) and perivascular interstitial cells of Cajal (PICC) in the rat bladder have been shown to display spontaneous Ca^2+^ oscillations [17]. PICC are also present in guinea-pig bladder [18] and their spontaneous activity was apparent in the current study (figure 7A, B). The profiles of Ca^2+^-oscillations differed between the two cell types with VSM frequency 1.85±0.15 min^−1^ (n = 45) and duration of 10.26±0.35 s (n = 114) in contrast to PICC which displayed significantly fewer (1.18±0.13 min^−1^, n = 21; P = 0.006, Mann-Whitney test) longer duration events (26.83±1.80 s, n = 50; P<0.001, Mann-Whitney test). Amplitude of Ca^2+^-oscillations was similar in the two cell populations, P = 0.168, Mann-Whitney (figure 6D).

**Figure 8:**
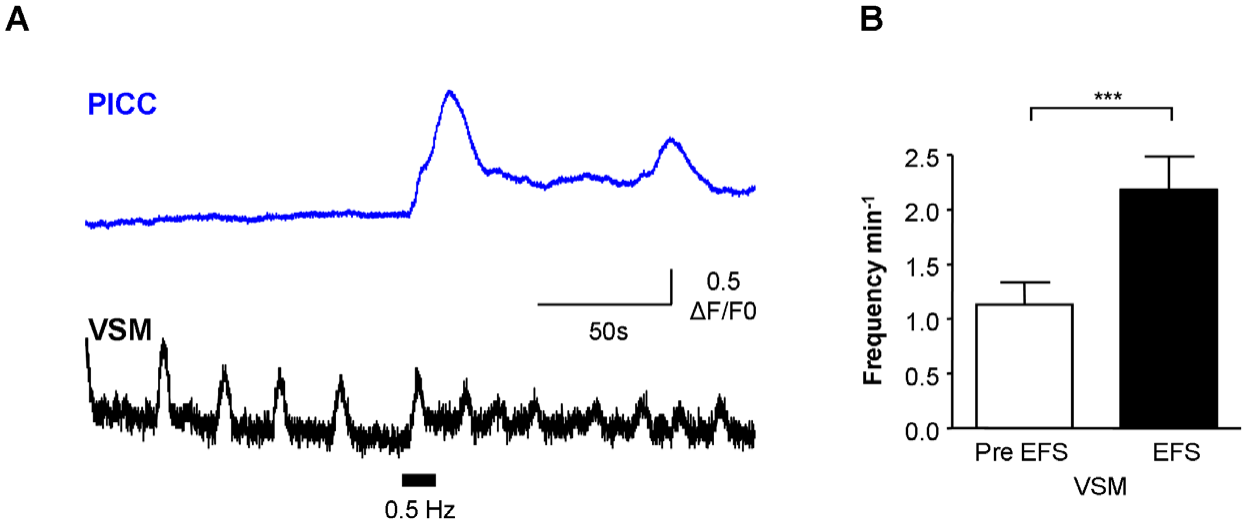
Responses of VSM and PICC to neurogenic stimulation. A, B. Neurogenic stimulation (EFS, 0.5 Hz) simultaneously evoked responses in PICC and VSM in the example shown. The PICC was quiescent pre-EFS but responded with several Ca^2+^-oscillations. The pre-EFS spontaneous activity in VSM was enhanced by 0.5 Hz. This was typical of 9/14 cells where EFS enhanced the frequency of VSM events as illustrated in the summary graph of panel G.

VSM and associated PICC responded simultaneously to EFS at 0.5 Hz (figure 8A) which resulted in an increase in Ca^2+^ oscillation frequency in VSM (typical of 9/14 preparations, P = 0.006, paired t-test). In the other 5 preparations, spontaneous oscillation frequency was unaffected by EFS, although the amplitude of the first oscillation during EFS tended to be larger. EFS evoked Ca^2+^-oscillations in previously quiescent PICC (typical of 4/9 preparations; mean amplitude 1.83±0.62 ΔF/F_0_) whereas in 5/9 preparations, EFS did not affect the frequency of spontaneous PICC Ca^2+^-oscillations.

## Discussion

The present study has provided novel evidence that bladder ICC respond to neurogenic stimulation, demonstrating their functional innervation and indicating that bladder ICC sub-populations are under direct control of the complex innervation that governs normal bladder function. Previous work on isolated bladder ICC [5], [6], [7] and ICC within tissue sheets [7], [19] demonstrated their expression of functional receptors. In addition, morphological and ultrastructural evidence has shown the physical relationship between ICC and intramural nerves [1], [14] to be as close as 20 nm. The experimental design of the present study is perhaps the only method of determining whether ICC are functionally innervated as it enabled direct visualisation of the cells responding to EFS in real time. The finding that EFS-evoked responses in ICC, which had been generated with pulses known to selectively stimulate nerves (pulse width 0.2 ms), were inhibited by TTX, confirmed their neurogenic origin. While we assume that the TTX inhibition is due to blockade of intramural nerves, we do not discount the possibility that TTX-sensitive Na+ channels may exist in bladder ICC. However, there is currently no evidence to support a current with the biophysical or pharmacological profile of a Na^+^ current in bladder ICC.

### Spontaneous and Neurogenic Ca^2+^-activity in ICC-LP

ICC-LP exhibited spontaneous Ca^2+^-oscillations at similar frequencies to that of ICC-IM but markedly less than that of bladder SMC. ICC-LP have been suggested to modulate detrusor smooth muscle spontaneous activity during filling; based on observations that removal of the mucosal layer from bladder strips reduced spontaneous activity [20] and experiments in transverse bladder sections which showed that spontaneous Ca^2+^/depolarizing activity originated in the lamina propria region before propagating to the detrusor [19]. One might expect putative modulatory, pacemaker-type cells to have a frequency of Ca^2+^-signals similar to (or greater) than the SMC, however, the SMC rate is more than 4 times that of ICC-LP. The fact that detrusor SMC have spontaneous electrical and Ca^2+^-activity indicates a myogenic origin [21], [22] however, it seems reasonable that detrusor SMC may be influenced by an excitatory input from the ICC-LP network.

Our finding that neurogenic stimulation evoked near-simultaneous responses in neighbouring ICC-LP whose activity had been non-synchronous pre-stimulation indicates the ability of nerves to exert higher control over the ICC-LP network. The observation here that spontaneous activity in ICC-LP (also ICC-IM, PICC, data not shown) was diminished in the presence of TTX suggests involvement of a basal, background neuronal activity. This is consistent with reports of spontaneous purinergic neurotransmission in mouse bladder where parasympathetic ATP release generated rises in Ca^2+^, depolarizations and action potentials in SMC [23]. In addition, the observed reduction in the amplitude of spontaneous oscillations during exposure to atropine may indicate a background level of acetylcholine from neuronal or non-neuronal sources which has been suggested by Zagorodnyuk et al. [24]. Previous reports that enzymatically dispersed ICC-LP and ICC-IM exhibit spontaneous Ca^2+^-activity in the absence of agonists [5], [6] would argue against their spontaneous activity being entirely explained by a neurogenic input.

### Spontaneous Ca^2+^-activity in ICC-IM and Neurogenic Stimulation

Detrusor ICC-IM are shown here to be functionally innervated by their responses to EFS-evoked neurogenic stimulation. ICC-IM spontaneous activity was not correlated with that of neighbouring SMC consistent with other studies [7], [25] however neurogenic stimulation remarkably synchronized the activity of both detrusor SMC and ICC-IM. If EFS-activity is analogous to bladder emptying under control of parasympathetic nerves, the spontaneous activity in both cell types, typical of the filling phase would be overridden, facilitating efficient, coordinated bladder emptying.

During filling, myogenic activity of detrusor smooth muscle may represent a balance of modulatory input from ICC-LP and ICC-IM populations. Transmitter release from parasympathetic intramural nerves is likely to be minimal at this stage, however we now know that the increased neuronal activity that stimulates voiding contractions will directly impact bladder ICC-LP, ICC-IM and detrusor SMC. Moreover, in diseased bladders, increased neurotransmitter release during filling will impact ICC populations in addition to SMC.

### PICC and Vascular Smooth Muscle

We recently reported novel PICC on guinea-pig bladder microvessels [18] and here show evidence of their neuronal regulation. Like ICC-IM and detrusor SMC, PICC spontaneous activity was less frequent than VSM, and the two cell types were apparently not synchronized. This contrasts with a recent investigation of rat bladder microvessels and their associated PICC where synchronous Ca^2+^-events occurred in PICC and VSM [17]. Comparison of the micrographs in the present study with those in Hashitani et al [17] suggests that different PICCs may have been studied; guinea-pig PICC were located on the boundary of smooth muscle-containing microvessels, presumably pre-capillary arterioles, whereas rat PICCs were a network overlying the microvessel which were described as post-capillary venules. The roles of PICC during bladder filling and emptying have yet to be elucidated but we propose that they may represent a fine-tuning mechanism for vascular tone and bladder perfusion in response to differing metabolic needs.

The findings do not appear to support a scenario in bladder where ICC act as intermediaries in transmission of signals from nerve to smooth muscle, as happens in gut [12], rather, SMC and ICC appear to be specifically targeted by bladder nerves. We have shown that ICC-IM and SMC respond apparently simultaneously to EFS implying that one cell was not an intermediary to the other, yet there remains the possibility that the ICC-IM EFS response is indirect, via neuronal activation of the smooth muscle and subsequent electrical or chemical signalling to the ICC. Interestingly, some ICC-LP exhibited a lag in responding to EFS, perhaps indicating that not all ICC-LP are directly innervated but that a sub-group of innervated ICC-LP respond to EFS with the delay representing transmission of the EFS response from an innervated cell across the network to other ICC-LP.

### Conclusions

The novel finding that bladder ICC sub-populations respond to EFS supports the hypothesis that they are functionally innervated and advances our knowledge of physiological communication between ICC and other cells within the bladder wall. Defects in signalling between nerves and ICC in the neurogenic bladder may be responsible for neurogenic bladder dysfunction and requires further investigation.
